# Supplementary material for: Oncolytic viruses engineered to enforce cholesterol efflux restore tumor-associated macrophage phagocytosis and anti-tumor immunity in glioblastoma
Source: Nat Commun. 2023 Jul 20;14:4367. doi: 10.1038/s41467-023-39683-z (PMC10359270; doi:10.1038/s41467-023-39683-z)
Supplement: Supplementary file 1 — Supplementary Information [file 41467_2023_39683_MOESM1_ESM.pdf]

Supplementary Information

- **SUPPLEMENTARY TABLE 1. CLINICAL DATA OF PATIENTS**
- **SUPPLEMENTARY TABLE 2. KEY RESOURCES TABLE**
- **SUPPLEMENTARY TABLE 3. ANTIBODY INFORMATION TABLE**
- **SUPPLEMENTARY FIGURES**
- **SUPPLEMENTARY NOTE 1. APPROVED CLINICAL STUDY PROTOCAL**

Supplementary Table 1 |. Clinical data of patients

Patient ID	Diagnosis	Molecular Classification	Reason of surgery
GBM-1 (15206607)	GBM	IDH1 WT, IDH2 WT, pTERT C228T C250T, pMGMT-met	Intracranial space occupying lesion
GBM-2 (15213895)	GBM	IDH1 WT, IDH2 WT, pTERT C250T, pMGMT-unmet	Intracranial space occupying lesion
GBM-3 (3340379)	GBM	IDH1 WT, IDH2 WT, pTERT WT, pMGMT-met	Borderline tumor of the temporal lobe
GBM-4 (15295799)	GBM	N/A	Intracranial space occupying lesion
GBM-5 (15308780)	GBM	IDH1 WT, IDH2 WT, pTERT C228T, pMGMT-unmet	Malignant brain tumor
GBM-6 (15329637)	GBM	IDH1 WT, IDH2 WT, pTERT WT, pMGMT-met	Intracranial space occupying lesion

Supplementary Table 2 |. Key resources table

REAGENT or RESOURCE	SOURCE	IDENTIFIER
**Chemicals, Peptides, and Recombinant Proteins**		
Filipin III	Cayman	480-49-9
NBD-cholesterol	Cayman	600441
24(S),25-epoxy Cholesterol	Cayman	10131
Cholesterol-water solubility	Sigma	C4951
Methyl-b-cyclodextrin	Sigma	C4555
Clodronate Liposomes	Yeasen	40337ES10
LXR-623	Glpbio	GC13954
U-18666A	MCE	HY-107433
DIDS sodium salt	MCE	HY-D0086
Hexidium Iodide	MCE	HY-114227
Propidium Iodide	MCE	HY-D0815
CC-5013	MCE	HY-A0003
DAPI	Beyotime	C1002
LPS	Beyotime	S1732
7-Ketocholesterol	TargetMol	TN6715
Doxycycline	Selleck	S5159
OVA peptide (257-264)	Sangon Biotech	T510212
Recombinant Murine IL-2	Sangon Biotech	C600128
Recombinant Human IL-6	Sangon Biotech	C610007
Recombinant Murine M-CSF	Sangon Biotech	C600234
Recombinant Human Apolipoprotein A-I	Sangon Biotech	C600421
Recombinant Human Tumor Necrosis Factor-alpha	Sangon Biotech	C600021
**Bacterial and Viruses**		
S. aureus	NISSUI	50231
Ad-GFP-LC3B	Beyotime	C3006
**Critical Commercial Assays**		
Cholesterol Extraction Kit	Sigma	MAK175
Amplex Red Cholesterol Assay Kit	Invitrogen	A12216
HDL and LDL/VLDL Quantification Kit	BioVision	K613
Cholesterol Efflux Fluorometric Assay Kit	BioVision	K582
D-Luciferin, Potassium Salt	BioVision	7903
Giemsa Staining	Beyotime	C0133
Annexin V-EGFP/PI Apoptosis Kit	Beyotime	C1067S
MTT Cell Proliferation and Cytotoxicity Assay Kit	Beyotime	C0009S
Cell mitochondrial Isolation Kit	Beyotime	C3601
Mito-Tracker Red CMXRos	Beyotime	C1035
Mitochondrial Membrane Potential Assay Kit with JC-1	Beyotime	C2003S
Fast Silver Dye Kit	Beyotime	P0017S
CFSE Cell Division Tracker Kit	Biolegend	423801
Cyto-Fast™ Fix/Perm Buffer Set	Biolegend	426803

Human Apolipoprotein A-I/ApoA1 ELISA	R&D	DY3664
ELISA MAX™ Deluxe Set Mouse IFN-γ	Biolegend	430804
AceQ Universal SYBR qPCR Master Mix	Vazyme	Q511-02
**Biological Samples**		
hPBMC	Milestone	MSZXS-202111
Glioma and Matching Paracarcinoma Tissue Chips	Bioaitech	N092Ct01
**Oligonucleotides**		
E1A-F: CCTTCTAACACACCTCCTGAGATACA	Genescript	N/A
E1A-R: CAGGCTCGTTAAGCAAGTCCTC	Genescript	N/A
Gapdh-F: AGGTCGGTGTGAACGGATTTG	Genescript	N/A
Gapdh-R: TGTAGACCATGTAGTTGAGGTCA	Genescript	N/A
Ears2-F: GGTTTTCTGCCAGAAGCCTTGC	Genescript	N/A
Ears2-R: TCTGGGAGCTTCTCTAGGTCCA	Genescript	N/A
Twnk-F: GTCTGCTGAAGGGACATCGGAA	Genescript	N/A
Twnk-R: GGCTAGTCTCACGTTGCTGATC	Genescript	N/A
Elac2-F: GAGGAGTATCGGAAGAACGTGC	Genescript	N/A
Elac2-R: GCTTAGGTTGACGAGTGTGGAAC	Genescript	N/A
Yars2-F: CATGTCTGGCTACGAGTTCATCC	Genescript	N/A
Yars2-R: TAGCCAAACCGCATTGCCAGCA	Genescript	N/A
Fastkd5-F: CTCAGCCTTACGCATCCTGGAT	Genescript	N/A
Fastkd5-R: TTCAGAGTTCCAAAGGACCACAG	Genescript	N/A
Tfam-F: GAGGCAAAGGATGATTCGGCTC	Genescript	N/A
Tfam-R: CGAATCCTATCATCTTTAGCAAGC	Genescript	N/A
Pnpt1-F: GCCTTCCCAATTCATGCCGTTG	Genescript	N/A
Pnpt1-R: CTCTGTCAGAGGAGCCAATCTC	Genescript	N/A
Gars-F: GATCCTGGAGATTGACTGCACC	Genescript	N/A
Gars-R: TCAACAGGTGGTCTGCTCGGAA	Genescript	N/A
Trmt10b-F: GAGAACGAAGGAAAGCCAAGCG	Genescript	N/A
Trmt10b-R: TGGTCCTGAGTGTTTGGCTTCC	Genescript	N/A
Tbrg4-F: GCTCGCTTTCAGCGACTTGTCA	Genescript	N/A
Tbrg4-R: ACTTCCTGCTCCACTGACTGCA	Genescript	N/A
Mrpl12-F: CTCAGACCTCAACGAACTCCTG	Genescript	N/A
Mrpl12-R: GGTGAAGTGTGTCCGCTCTTTC	Genescript	N/A
Tefm-F: AGTCACAGGAGAAGTGGCTGCT	Genescript	N/A
Tefm-R: GGTCAAGGTGAGCCCATGCAAT	Genescript	N/A
Trmt5-F: CTGAATCTCCGAGATCACCAGC	Genescript	N/A
Trmt5-R: TTCCTCTCCACACAGCACTTCC	Genescript	N/A
Trnt1-F: ACATGGGACTATCACTGCCAGG	Genescript	N/A
Trnt1-R: CTTCGTTCAGCGTCTTTCTGCC	Genescript	N/A
Trmt10c-F: CATAGCAACAGAGTGCCTTCCAC	Genescript	N/A
Trmt10c-R: CCTGGGAACAAACTTGAGAGCC	Genescript	N/A
**Recombinant DNA**		
pTNF-α-promoter-luc	Beyotime	D2480

pNFκB-luc	Beyotime	D2206
pSTAT3-TA-luc	Beyotime	D2259
pENTR™/D-TOPO®	Invitrogen	K2400-20
pAd/PL-DEST™ Gateway	Invitrogen	V49420
pLenti-EF1a-YFP-P2A-Puro	OBIO	N/A
pLenti-EF1a-YFP-P2A-Puro-CMV-hAPOA1	OBIO	N/A
pLenti-EF1a-Puro-CMV-mCAR-3Flag	OBIO	N/A
pLenti-SV40-Luciferase-IRES-Puro	Genechem	N/A
pLenti-SV40-Luciferase-IRES-Puro-CMV-OVA	Genechem	N/A
pLenti-SV40-Luciferase-IRES-Puro-CMV-mCAR-3Flag	Genechem	N/A

Contact for reagent and resource sharing

Further information and requests for resources and reagents should be directed to and will be fulfilled by the Lead Contact, Jiwu Wei (wjw@nju.edu.cn).

Supplementary Table 3 |. Antibody information table

Antibodies	Source	Identifier	Dilution ratio
Anti-ApoE (EPR19392)	Abcam	ab183597	1:50
Anti-Hexon	Abcam	ab252760	1:50
Anti-CD16/CD32 (2.4G2)	BD Pharmingen	553142	1:100
Anti-ABCA1 (5A1-1422)	BIO-RAD	MCA2681	1:100
Anti-OVA (1D3D5)	Proteintech	67614-1-Ig	1:1000
Anti-CD45 (30-F11)	Biolegend	103112	1:100
Anti-CD45 (HI30)	Biolegend	304011	1:100
Anti-CD45.1 (A20)	Biolegend	110706	1:100
Anti-CD45.2 (104)	Biolegend	109814	1:100
Anti-CD11b (M1/70)	Biolegend	101206	1:100
Anti-CD11b (ICRF44)	Biolegend	301329	1:100
Anti-F4/80 (BM8)	Biolegend	123126	1:100
Anti-CD3 (17A2)	Biolegend	100204	1:100
Anti-CD4 (GK1.5)	Biolegend	100408	1:100
Anti-CD8 (53-6.7)	Biolegend	100732	1:100
Anti-NK1.1 (S17016D)	Biolegend	156508	1:100
Anti-CD36 (HM36)	Biolegend	102606	1:100
Anti-CD64 (S18017D)	Biolegend	161004	1:100
Anti-CD68 (Y1/82A)	Biolegend	333813	1:100
Anti-PD-1 (29F.1A12)	Biolegend	135206	1:100
Anti-PD-1 (EH12.2H7)	Biolegend	329905	1:100
Anti-PD-L1 (10F.9G2)	Biolegend	124308	1:100
Anti-Siglec-g (Siglec-10, SH1)	Biolegend	163302	1:100
Anti-Siglec-10 (5G6)	Biolegend	347603	1:100
Anti-SIRPα (P84)	Biolegend	144012	1:100
Anti-LAG-3 (C9B7W)	Biolegend	125208	1:100
Anti-TIGIT (4D4/mTIGIT)	Biolegend	156104	1:100
Anti-I-A/I-E (MHC-I, M5/114.15.2)	Biolegend	107608	1:100
Anti-I-A^b (MHC-II, AF6-120.1)	Biolegend	116408	1:100
Anti-TNF-α (MP6-XT22)	Biolegend	506306	1:100
Anti-Nos2 (iNOS, W16030C)	Biolegend	696806	1:100
Anti-IFN-γ (XMG1.2)	Biolegend	505808	1:100
Anti-Gzm-B (QA16A02)	Biolegend	372208	1:100
Anti-XBP1 s (9D11A43)	Biolegend	658802	1:1000
Anti-CD44 (IM7)	Biolegend	103007	1:100
Anti-CD62 L (MEL-14)	Biolegend	104406	1:100
Anti-FOXP3 (MF-14)	Biolegend	126404	1:100
Alexa Fluor® 488 Donkey anti-rabbit IgG	Biolegend	406416	1:100
InVivo Mab rat IgG1 Isotype control (TNP6A7)	BioXCell	BP0290	10 mg/kg
InVivo Mab Anti-mouse CD4 (GK1.5)	BioXCell	BE0003-1	10 mg/kg
InVivo Mab Anti-mouse CD8α (YTS 169.4)	BioXCell	BE0117	10 mg/kg

InVivo Mab Anti-mouse NK1.1 (PK136)	BioXCell	BE0036	10 mg/kg
InVivo Mab Anti-mouse CSF1R (AFS98)	BioXCell	BE0213	10 mg/kg
Anti-SREBP2	Proteintech	28212-1-AP	1:1000
Anti-LDLR	Proteintech	10785-1-AP	1:50 (IF), 1:1000 (WB)
Anti-β-Actin (2D4H5)	Proteintech	66009-1-Ig	1:1000
Anti-ApoA1	Proteintech	14427-1-AP	1:1000
Anti-ABCG1	Proteintech	13578-1-AP	1:100 (FC), 1:1000 (WB)
Anti-NeuN	Servicebio	GB11138	1:50
Anti-GFAP	Servicebio	GB11096	1:50
Anti-EGFR	Sangon Biotech	D160292	1:1000
Anti-ABCA1 (HJ1)	Abcam	ab66217	1:100 (FC), 1:1000 (WB)
Anti-F4/80 MicroBeads UltraPure, mouse	Miltenyi Biotec	130-110-443	1:10
CD11b MicroBeads UltraPure, mouse	Miltenyi Biotec	130-126-725	1:10
CD8a (Ly-2) MicroBeads, mouse	Miltenyi Biotec	130-117-044	1:10
CD14 MicroBeads, human	Miltenyi Biotec	130-050-201	1:10
T-Cell Activation/Expansion Kit, mouse	Miltenyi Biotec	130-093-627	bead-to-cell ratio 1:1

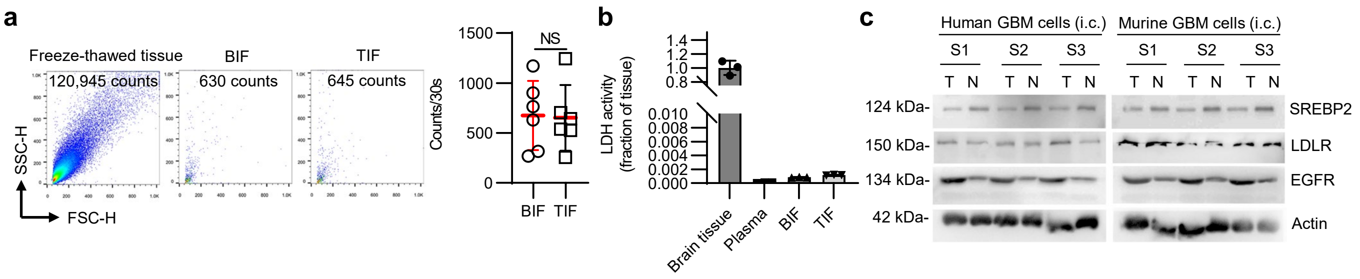

Supplementary Figure 1 |. Data related to Figure 1

(**a**) Representative flow plots and quantification of cell debris in BIF and TIF. The collected interstitial fluid was recorded by flow cytometry for 50 s to assess the level of debris. n = 6 biological samples per group. (**b**) Quantification of LDH activity in brain tissue, plasma, BIF and TIF. Equal volumes of plasma, BIF, and TIF were used for the LDH assay, and their values were normalized to BIF-separated brain tissue lysates. n = 3 biological samples per group. (**c**) Western blot showing the expression levels of SREBP2, LDLR, and EGFR in brain tissue samples (n = 3) and tumor tissue samples (n = 3) from intracranial U138-MG (left) and GL261 (right) models. Statistical significance was determined using the Mann–Whitney test (Two-tailed) in **a**. Data shown are the mean ± SD. Source data are provided in the Source Data file.

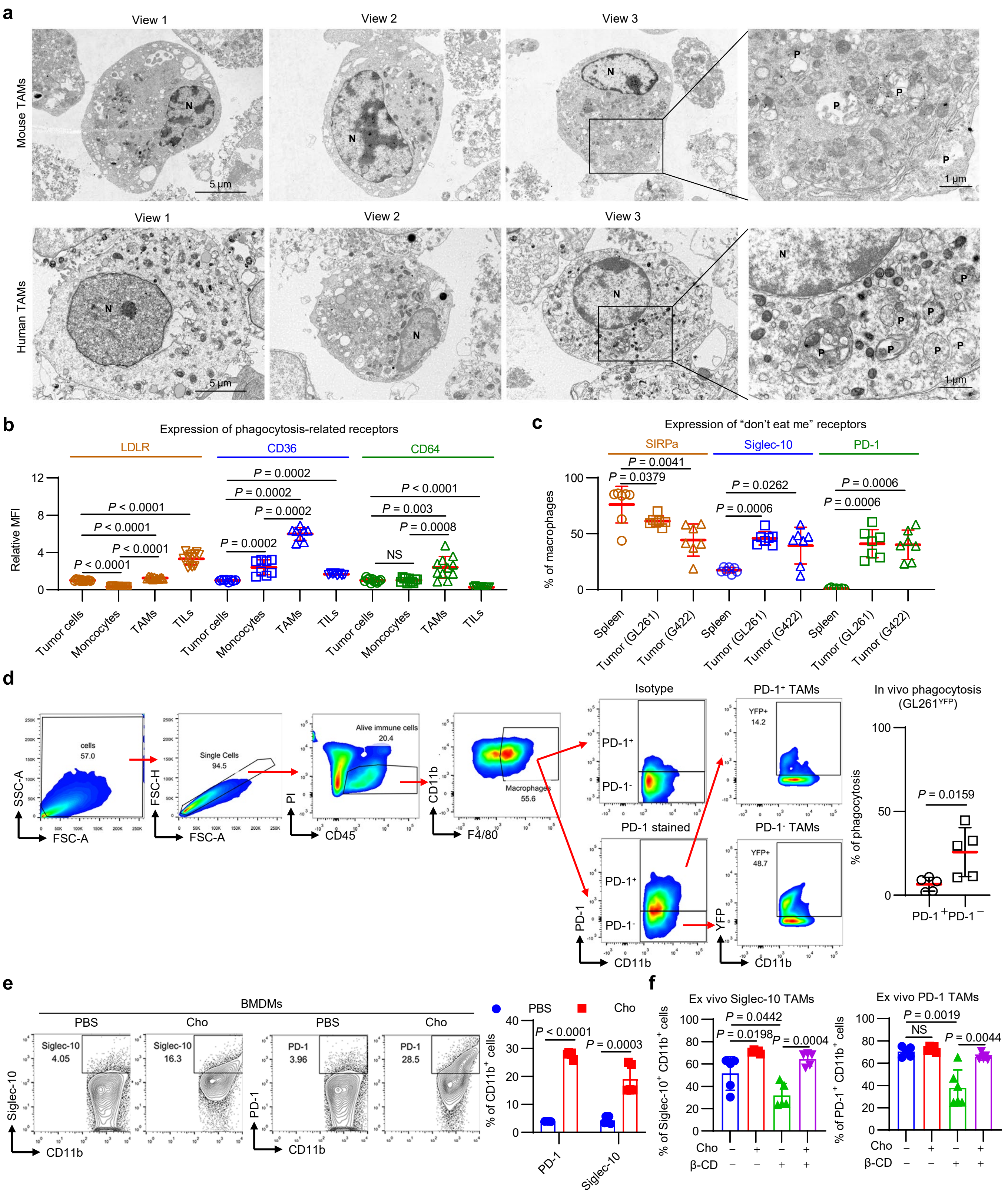

a
View 1
View 2
View 3
Mouse TAMs
Human TAMs
N
P
5 μm
1 μm
b
Expression of phagocytosis-related receptors
LDLR
CD36
CD64
Relative MFI
P < 0.0001
P = 0.0002
P = 0.003
P = 0.0008
NS
Tumor cells
Moncocytes
TAMs
TILs
c
Expression of "don't eat me" receptors
SIRPa
Siglec-10
PD-1
P = 0.0041
P = 0.0379
P = 0.0262
P = 0.0006
% of macrophages
Spleen
Tumor (GL261)
Tumor (G422)
d
cells 57.0
Single Cells 94.5
Alive immune cells 20.4
Macrophages 55.6
SSC-A
FSC-A
FSC-H
PI
CD45
CD11b
F4/80
Isotype
PD-1 stained
PD-1+ TAMs
PD-1- TAMs
YFP+ 14.2
YFP+ 48.7
PD-1
YFP
In vivo phagocytosis (GL261YFP)
P = 0.0159
% of phagocytosis
PD-1+
PD-1−
e
BMDMs
PBS
Cho
Siglec-10 4.05
Siglec-10 16.3
PD-1 3.96
PD-1 28.5
Siglec-10
CD11b
P < 0.0001
P = 0.0003
% of CD11b+ cells
f
Ex vivo Siglec-10 TAMs
Ex vivo PD-1 TAMs
P = 0.0442
P = 0.0198
P = 0.0004
P = 0.0019
P = 0.0044
% of Siglec-10+ CD11b+ cells
% of PD-1+ CD11b+ cells
Cho
β-CD

Supplementary Figure 2 |. Data related to Figure 2

(**a**) Representative TEM images of mouse and human TAMs. TAMs were isolated from intracranial GL261 tumors or GBM patient tissues. These macrophages had prominent cellular inclusions, including multiple non-cleared phagosomes (P). Their nuclear chromatin (N) is rich in tightly packed regions (darker). The images are representative of 2 samples per groups. (**b**) Quantification of cell subpopulations in orthotopic GBMs and the expression levels of sterol uptake receptors. Intracranial GL261-bearing C57BL/6J mice were sacrificed on day 17 after tumor inoculation. Tumor cell, monocyte, macrophage, and lymphocyte subpopulations were determined as described above. The expression of LDLR, CD36, and CD64 was analyzed by flow cytometry. n = 12 animals per group. (**c**) Quantification of "don't eat me" receptor expression on peripheral macrophages and TAMs. Intracranial GL261-bearing C57BL/6J mice and G422-bearing KM mice were sacrificed on day 17 after tumor inoculation. Splenic macrophages and TAMs were analyzed for SIRPa, Siglec-10, and PD-1 expression by flow cytometry. n = 7 animals per group. (**d**) Representative flow plots and quantification of in vivo phagocytosis by $PD1^+$ TAMs and $PD1^-$ TAMs. Intracranial $GL261^{YFP}$-bearing C57BL/6J mice were sacrificed on day 17 after tumor inoculation. TAMs were examined for PD1 expression and YFP signals. n = 5 animals per group. (**e**) Quantification of "don't eat me" expression in cholesterol-treated BMDMs. In vitro differentiated BMDMs were cultured with vehicle medium or Cho medium (10 µg/ml) for 24 h. The expression of Siglec-10 and PD-1 in the cells was determined by flow cytometry. n = 5 animals per group. (**f**) Quantification of "don't eat me" expression in cholesterol-modulated TAMs. Sorted TAMs were cultured with vehicle, or Cho (10 µg/ml) medium for 24 h; and were cultured with β-CD (0.5 mM) medium for 2 h. These TAMs were harvested and tested for the expression of Siglec-10 and PD-1. n = 5 animals per group. Statistical significance was determined using the Mann–Whitney test (Two-tailed) in **b**, **c**, **d** or the unpaired t test (Two-tailed) in **e**, **f**. Data shown are the mean ± SD. Source data are provided in the Source Data file.

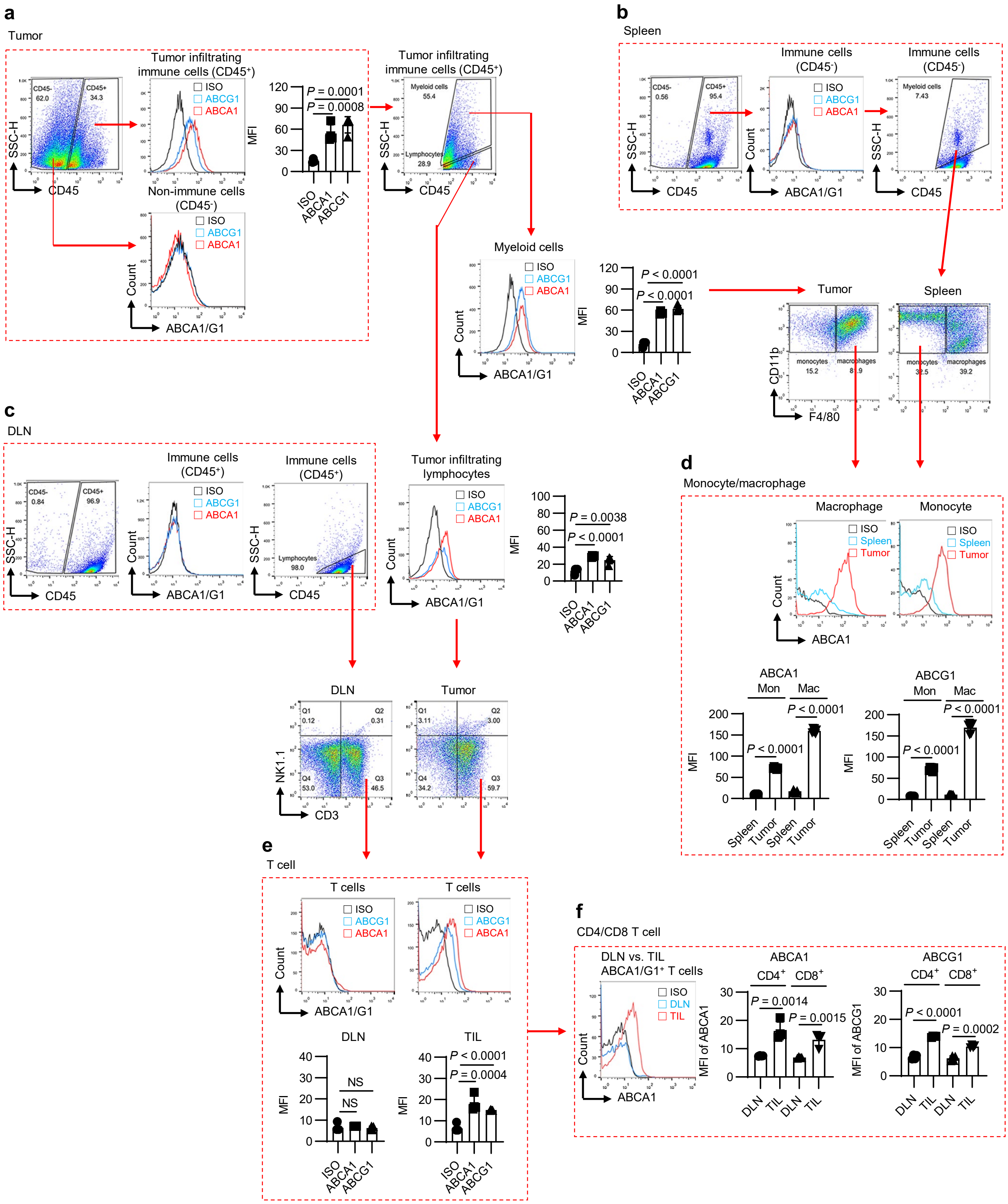

Supplementary Figure 3 |. Expression Distribution of Cholesterol Efflux Receptors (related to Figure 3)

In vivo expression analysis of ABCA1/G1 in intracranial GL261-bearing C57BL/6J mice. Tumor tissues, draining lymph nodes (DLNs), and spleen tissues were collected for analysis of ABCA1/G1 expression levels among different cell subsets, including tumor cells (**a**), splenocytes (**b**), draining lymph nodes (**c**), monocytes/macrophages (**d**), TILs (**e**), and CD4⁺/CD8⁺ T cells (**f**). Tumor, n = 4 samples per group. Spleen, n = 6 samples per group. DLN, n = 4 samples per group. Monocyte/macrophages, n = 6 samples per group. T cell, n = 4 samples per group. CD4⁺/CD8⁺ T cells, n = 4 samples per group. Statistical significance was determined using the unpaired t test (Two-tailed) in **a**, **b**, **c**, **d**, **e**, **f**. Data shown are the mean ± SD. Source data are provided in the Source Data file.

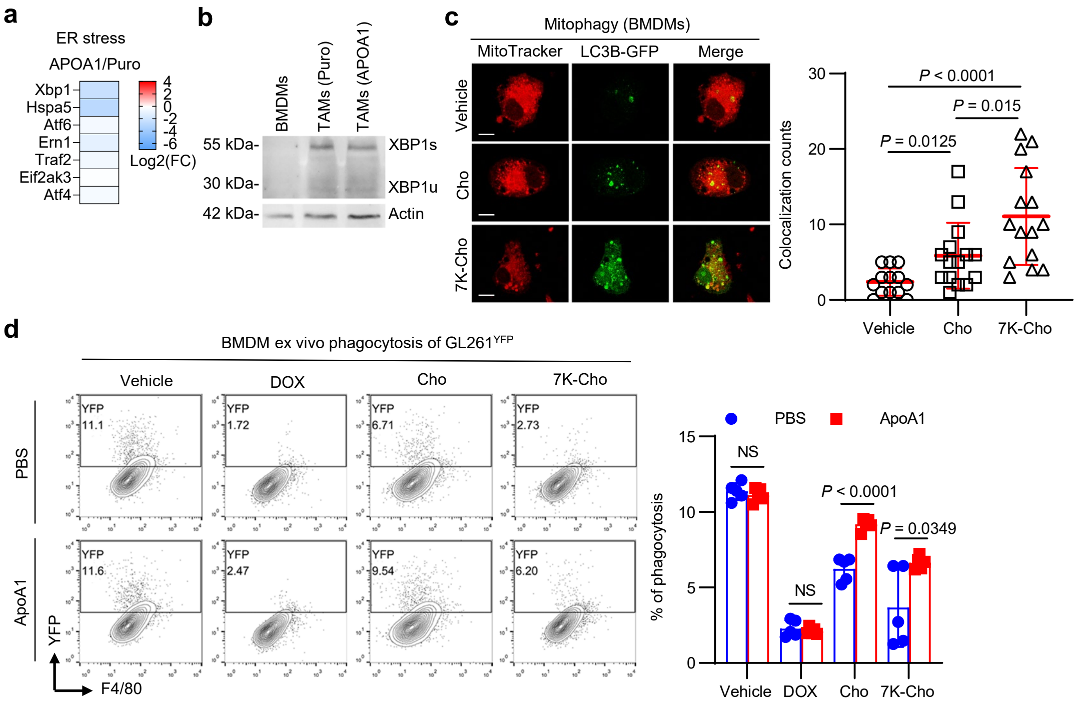

Supplementary Figure 4 |. 7-Ketocholesterol Induced Mitochondrial Stress in Macrophages (related to Figure 5)

(**a**) Heatmap of differentially expressed ER stress genes between Puro-TAMs and APOA1-TAMs. (**b**) Protein expression analysis of the ER stress sensor XBP1 in Puro-TAMs and APOA1-TAMs. n = 2 independent experiments. (**c**) Representative confocal images and quantification of mitophagy in cholesterol-treated BMDMs. BMDMs were cultured with vehicle, cholesterol (10 µg/ml), or 7-ketocholesterol (10 µg/ml) medium for 24 h. Mitophagy of these cells was determined by the colocalization (yellow) of mitochondria (red) with autophagosomes (green dots). Vehicle, n = 13. Cho, n= 15. 7K-Cho, n =15. Scale bar, 10 µm. (**d**) Quantification of ex vivo phagocytosis in BMDMs. BMDMs were cultured with vehicle, DOX (10 µg/ml), Cho (10 µg/ml), or 7K-Cho (10 µg/ml) medium in the absence or presence of ApoA1 (10 µg/ml) for 24 h. These BMDMs were harvested and cocultured with GL261^{YFP} cells in a U-bottom ultralow attachment 96-well plate for 4 h. Phagocytosis was determined by flow cytometry. n = 5 biological samples per group. Statistical significance was determined using the unpaired t test (Two-tailed) in **c**, **d**. All data are the mean ± SD. Source data are provided in the Source Data file.

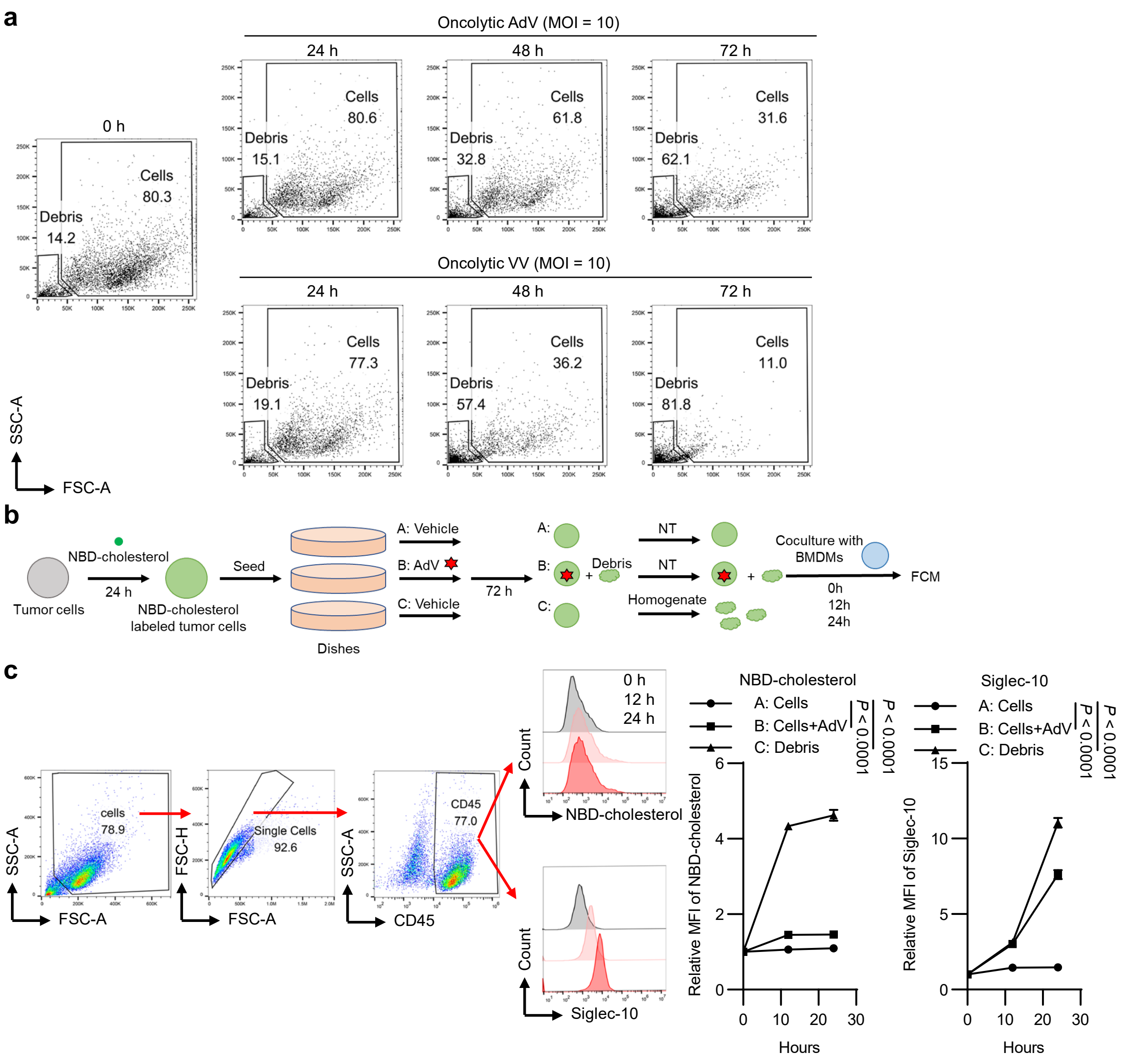

Supplementary Figure 5 |. Oncolysis of Oncolytic Viruses Promotes Macrophage Cholesterol Accumulation and Induces Phagocytic Fragility (related to Figure 7)

(**a**) Oncolysis of oncolytic viruses increases tumor debris production. Oncolytic AdV or VV (MOI=10) infected GL261 cells for 0, 4, 12, and 24 hours. Cell and supernatant were harvested for FCM analysis. AdV indicates adenovirus. VV indicates vaccinia virus. n = 2 independent experiments. (**b**) Experimental setup to investigate the effect of oncolysis-induced cell debris on phagocytosis and cholesterol internalization of macrophages. NT indicates untreated. (**c**) Quantification of cholesterol, phagocytosis, and Siglec-10 levels of BMDMs. n = 3 biological samples per group. Statistical significance was determined using the unpaired t test (Two-tailed) in **c**. All data are the mean ± SD. Source data are provided in the Source Data file.

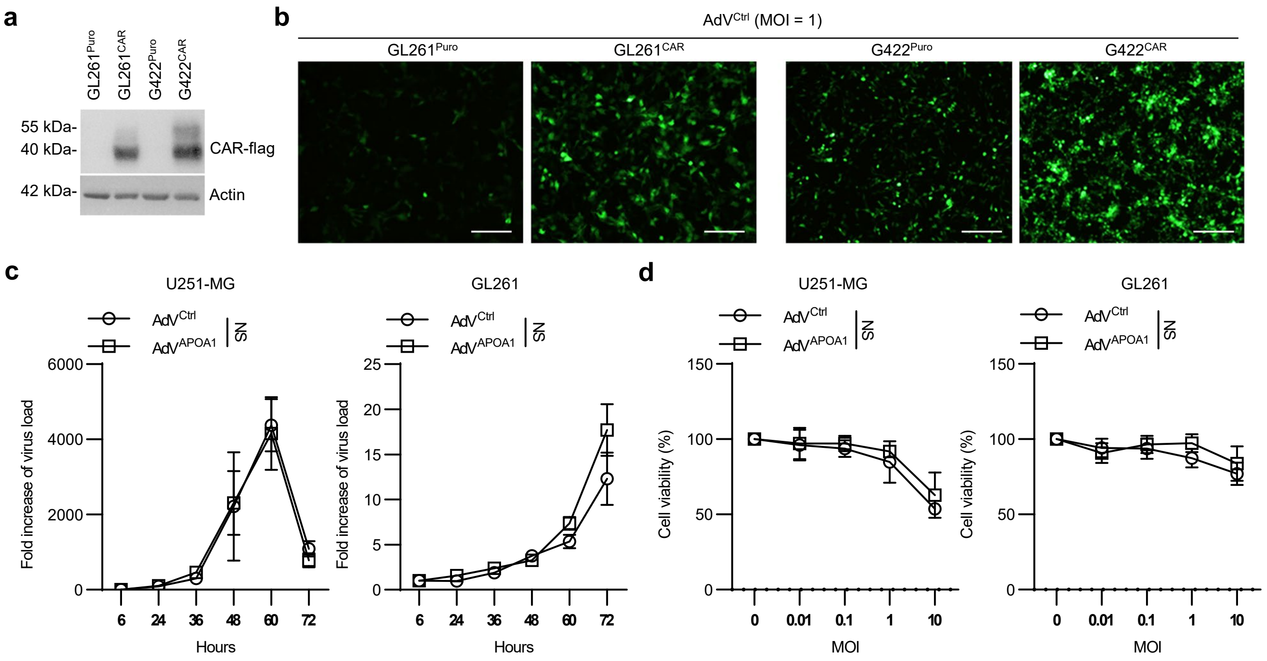

Supplementary Figure 6 |. Replication and Oncolysis of Recombinant Oncolytic Adenoviruses Expressing APOA1 (related to Figure 7)

(**a-b**) Western blot detection (**a**) and AdV^{Ctrl} infection (**b**) of murine GBM cells stably expressing murine coxsackievirus and adenovirus receptors (CAR). n = 2 independent experiments. Scale bar, 200 μm. (**c**) Replication of AdV^{APOA1} in GBM cell lines in vitro. GBM cell lines, U251-MG and GL261 were infected with OVs (MOI = 10) for 6, 24, 36, 48, 60, and 72 h. Cells were harvested, and virus copy number was determined by $TCID_{50}$. n = 3 independent experiments. (**d**) Oncolysis of AdV^{APOA1} in GBM cell lines in vitro. The four GBM cell lines were plated in 96-well plates (1 × 10^4 cells/well). Cells were cultured with the indicated MOI OVs for 72 h. Cell viability was determined by MTT assay. n = 3 independent experiments. Statistical significance was determined using the two-way ANOVA with Sidak's test in **c**, **d**. Data shown are the mean ± SD. Source data are provided in the Source Data file.

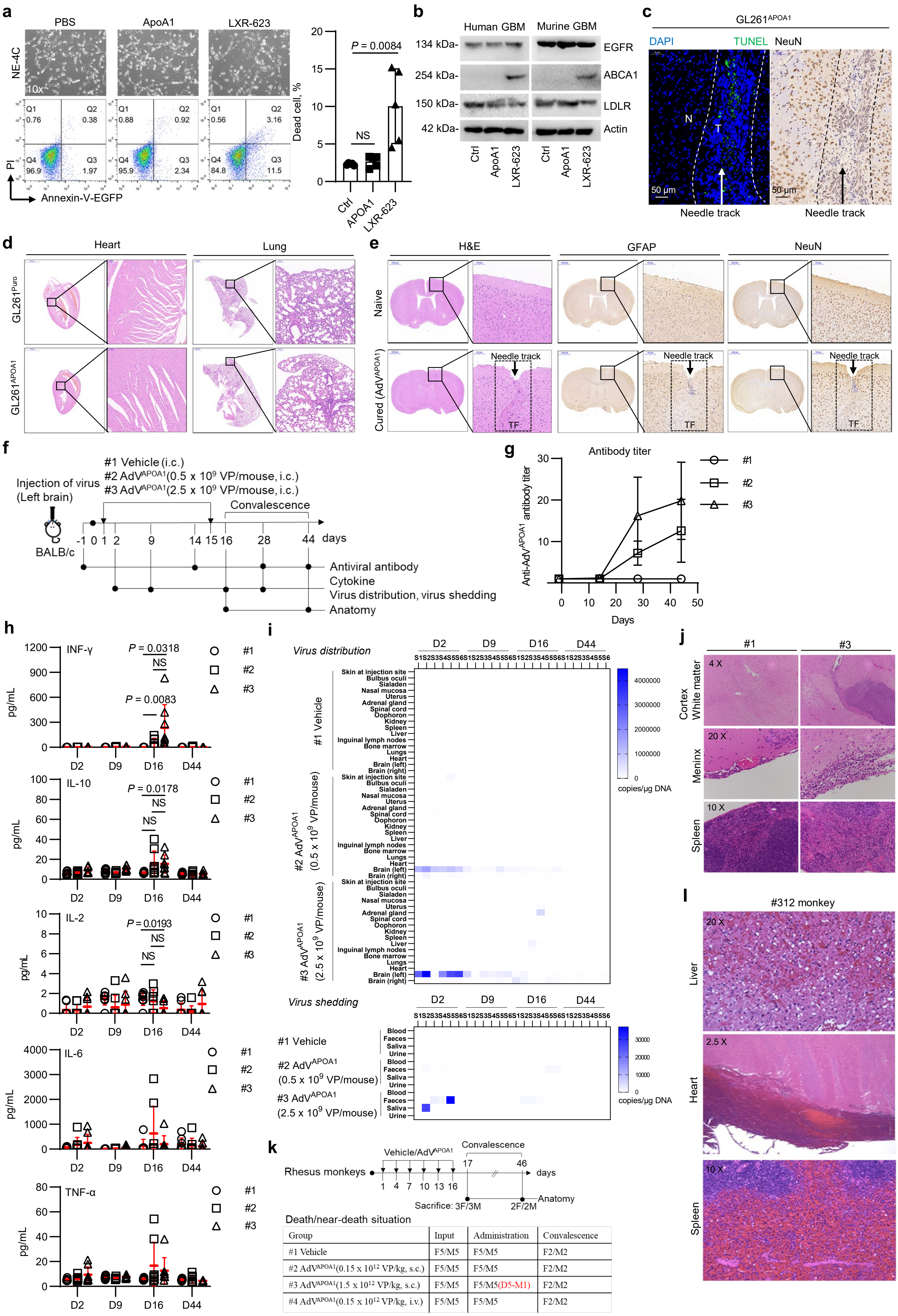

Death/near-death situation

Group	Input	Administration	Convalescence
#1 Vehicle	F5/M5	F5/M5	F2/M2
#2 AdV[APOA1](0.15 x 10[12] VP/kg, s.c.)	F5/M5	F5/M5	F2/M2
#3 AdV[APOA1](1.5 x 10[12] VP/kg, s.c.)	F5/M5	F5/M5(D5-M1)	F2/M2
#4 AdV[APOA1](0.15 x 10[12] VP/kg, i.v.)	F5/M5	F5/M5	F2/M2

Supplementary Figure 7 |. Preclinical Safety Evaluation of Local Administration of Oncolytic AdV^{APOA1} (related to Figure 7)

(**a**) Comparison of neurotoxic effects of LXR-623 and ApoA1 treatments. n = 5 independent experiments. (**b**) Western blot showing the expression levels of EGFR, ABCA1, and LDLR in ApoA1-treated GBM cells. U138-MG (left) and GL261 (right) cell lines were cultured with vehicle, ApoA1 (10 µg/ml), or LXR-623 (5 µM) in 1% FBS medium for 24 h. The cells were harvested for analysis of the indicated protein levels. n = 2 independent experiments. (**c**) Representative immunofluorescence and histochemical images of brain tumor tissues. TUNEL, TdT-mediated dUTP Nick-End Labelling. NeuN, neuronal nuclei. T, tumor; N, normal. The images are representative of 2 tumors. (**d**) Representative H&E staining of heart and lung tissues. The images are representative of 2 samples per group. (**e**) Representative H&E and histochemical staining of brain tissues. Slides were from naïve and AdV^{APOA1}-cured GL261$^{CAR\text{-}luc}$ tumor-bearing mice. GFAP, glial fibrillary acidic protein. TF indicates healing tumor focus. The images are representative of 2 samples per group. (**f**) A long-term toxicity study of intracerebral injection of AdV^{APOA1} in BALB/c mice. n = 64 animals per group. (**g**) Quantification of anti-AdV^{APOA1} antibody titers. n = 10 animals per group. (**h**) Quantification of cytokines. n = 8 animals per group. (**i**) Virus distribution and shedding studies. qPCR for quantitative analysis of viral genome copies. n = 24 animals per group. (**j**) Representative H&E staining of the brain (injection side) and spleen. Samples were from taken the end of dosing (D16). n = 8 animals per group. (**k**) A long-term toxicity study of subcutaneous injection or intravenous injection of AdV^{APOA1} in rhesus monkeys. On D5 of the administration period, one male monkey in the subcutaneous high-dose group died, and the rest of the tested animals survived until the planned dissection. F, female; M, male. n = 10 animals per group. (**l**) Representative H&E staining of the liver, heart, and spleen. Samples were obtained from dead monkey #312. n = 1. Statistical significance was determined using the unpaired t test (Two-tailed) in **a**, **h**. Data shown are the mean ± SD. Source data are provided in the Source Data file.

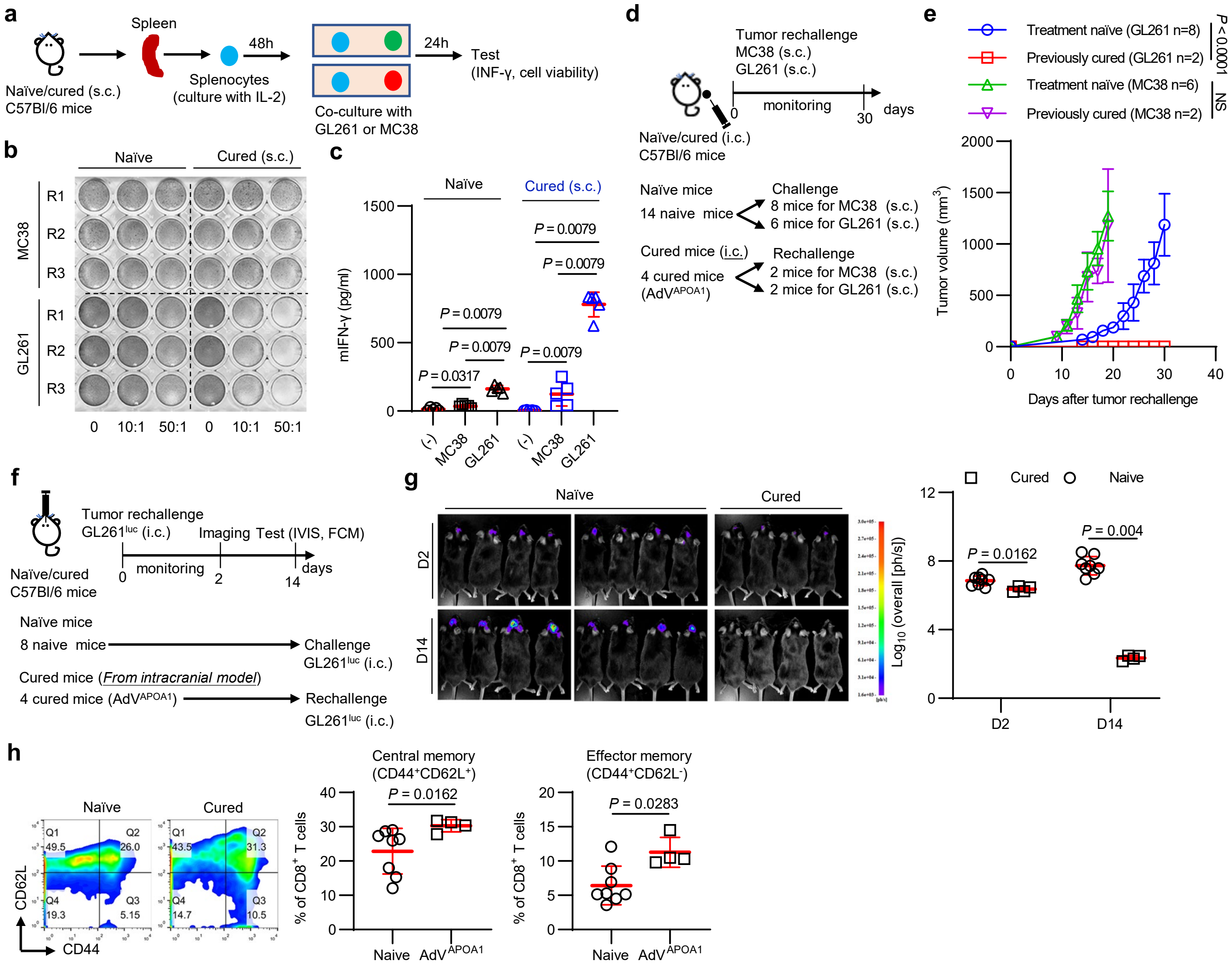

Supplementary Figure 8 |. Intratumoral Administration of Oncolytic AdV^{APOA1} Establishes Tumor-Specific Immune Memory (related to Figure 8)

(**a-c**) Experimental setup for tumor-specific killing (**a**), crystal violet staining (**b**), and IFN-γ production (**c**) of T-cells. Data are related to the experimental data (Figure 8g). Splenocytes from naïve mice or subcutaneously GL261 cured mice were stimulated with IL-2 (10 ng/ml) for 5 days to obtain activated T-cells. These T-cells were cocultured with MC38 cells or GL261 cells in 96-well plates for 24 h. Crystal violet staining was performed to determine tumor cell killing. 3 replicate wells per group. n = 2 independent experiments. ELISA was performed to identify IFN-γ secretion in the coculture supernatant. Experiments were conducted once. n = 5 biological samples per group. (**d-e**) Experimental setup (**d**) and tumor growth curves (**e**) of the subcutaneous tumor-rechallenge model. Naïve mice or i.c. cured mice were s.c. inoculated with 1 × 10^6 MC38 cells and 1 × 10^6 GL261 cells. Previously cured, n = 2 animals per group. Treatment naïve, n = 8 animals per group. (**f-g**) Experimental setup (**f**) and tumor growth quantification (**g**) of CD8$^+$ T cells in the intracranial tumor-rechallenge GBM model. Naïve mice and 90-day AdV^{APOA1}-cured mice were inoculated i.c. with 5 × 10^5 GL261^{luc} cells, respectively. IVIS was performed to determine tumor growth on days 2 and 14 after tumor rechallenge. Cured, n = 4 animals per group. Naïve, n = 8 animals per group. (**h**) Quantification of memory CD8$^+$ T cells in the intracranial tumor-rechallenge GBM model. Splenic CD8$^+$ T cells were analyzed for CD44 and CD62 L expression at the endpoint. Cured, n = 4 animals per group. Naïve, n = 8 animals per group. Statistical significance was determined using the Mann–Whitney test (Two-tailed) in **c**, **g**, **h**, or the log-rank test in **e**. Data shown are the mean ± SD. Source data are provided in the Source Data file.

Supplementary Note 1

Approved clinical study protocol

1. Research background

glioblastoma (GBM) is the most common primary intracranial tumor. The prognosis of patients with glioblastoma is poor. The malignant degree of glioblastoma is the highest and the median survival of patients is less than two years. At present, the main treatment of glioma is surgical resection, combined with radiotherapy, chemotherapy and other comprehensive treatment methods. There is no standard effective treatment for recurrent glioma. Elucidating the mechanism of the occurrence and development of glioma is crucial for finding new and effective treatment options.

Unlike other extracranial solid tumors, gliomas have unique anatomic and metabolic characteristics, resulting in a unique tumor metabolic and immune microenvironment. Previous studies have shown that glioma cells take up significantly more extracellular cholesterol than normal astrocytes. Glioblastoma can inhibit de novo synthesis of cholesterol, reduce ABCA1 expression and its mediated cholesterol efflux, upregulate LDLR to increase extracellular cholesterol uptake, and obtain a rich and continuous source of cholesterol. However, the remodeling of cholesterol metabolism in glioma still needs to be further elucidated, and the disorder of cholesterol metabolism is closely related to the remodeling of immune environment, and the role and mechanism of cholesterol metabolism on the immune microenvironment of glioma still need to be further elucidated.

Tumor-associated macrophages (TAMs) are the most abundant infiltrating immune cells in glioma tissue, accounting for 30-50% of all cells. High levels of cholesterol in glioma interstitial fluid lead to abnormal accumulation of cholesterol in TAMs. However, how does the abnormal accumulation of TAMs cholesterol contribute to the glioma immune microenvironment? What is the mechanism by which the immune microenvironment is regulated? Further clarification is needed. Increasing the efflux of cholesterol is an option to reduce abnormal cholesterol accumulation in glioma TAMs. Studies have shown that although macrophages can achieve cholesterol effection through their own production of apoE, apoA1 can increase apoE and ABCA1-mediated cholesterol effection.

This project will test the scientific hypothesis that cholesterol promotes glioma progression by regulating TAMs and thus T-cell function (cholesterol-TAMS-T cell regulatory axis). The enrichment of cholesterol in the glioma microenvironment leads to abnormal accumulation of cholesterol in tumor-associated macrophages and the increase of its metabolite 7-ketocholesterol, which leads to damage of phagocytic function of TAMs and inhibits its activation of T lymphocytes for tumor killing, thus promoting the progression of glioma. Apoa1-mediated cholesterol effection can be targeted to restore the cholesterol metabolic homeostasis of TAMs without affecting the cholesterol metabolism of other normal nerve cells, thus restoring the phagocytic function of TAMs and its immune activation to T lymphocytes, and inhibiting the progression of glioma.

2. Research purpose

Effect of cholesterol effection regulation on the phagocytic function of TAMs and anti-tumor immunity of T lymphocytes in patients with GBM tumor.

3. Research design and methods:

3.1 Scheduling Standards

Patients with GBM diagnosis and indications for surgery.

3.2 Research Design

The tumor tissue samples of 5-6 patients undergoing GBM surgery were collected. The TAMs were sorted by immunomagnetic beads, and the cytoplasmic phagosomes of TAMs were detected by transmission electron microscopy. The selected TAMs were divided into two groups, control group and cholesterol deprivation group, which were co-cultured with GFP-GBM cells, and the phagocytosis of TAMs was detected by flow cytometry. Five cases of GBM surgical tumor tissue samples were obtained, and the tumor tissues were transplanted by PDX. The tumorigenic mice were divided into control group and cholesterol deprivation group. The tumor progression was observed, and tumor volume and survival were recorded. Some tumor tissues were digested as single-cell suspensions, and the expression of TAMs cholesterol and phagocytic inhibitory receptors SIGLOC-10 and PD-1 was detected by flow cytometry.

3.3 Protection of vulnerable groups

The subjects of this study include glioma patients, and the following principles will be followed during the trial:

1. The research design and operation shall comply with relevant ethical requirements and regulations;
2. The inclusion of patients shall be signed and approved by the legal representative

3. Tumor resection for enrolled patients was required for routine clinical treatment, and no additional operations were required for this study.

4. Sample size calculation

Partial tumor tissue samples removed surgically from 5 patients

5. Data management and confidentiality

All records relating to the subject's identity will be kept confidential and will not be disclosed to the public except as permitted by applicable laws and/or regulations

6. Informed consent

The subject must obtain written informed consent before performing the study procedure, and inform the patient of the specific content related to the study, as detailed in informed consent.

7. Adverse event reporting

This study did not increase the number of additional adverse events in patients outside the usual clinical treatment.
