# Supplementary material for: Oncolytic viruses engineered to enforce cholesterol efflux restore tumor-associated macrophage phagocytosis and anti-tumor immunity in glioblastoma
Source: Nat Commun. 2023 Jul 20;14:4367. doi: 10.1038/s41467-023-39683-z (PMC10359270; doi:10.1038/s41467-023-39683-z)
Supplement: Supplementary file 3 — Reporting Summary [file 41467_2023_39683_MOESM3_ESM.pdf]

nature portfolio

Corresponding author(s): Jiwu Wei

Last updated by author(s): June 15, 2023

Reporting Summary

Nature Portfolio wishes to improve the reproducibility of the work that we publish. This form provides structure for consistency and transparency in reporting. For further information on Nature Portfolio policies, see our Editorial Policies and the Editorial Policy Checklist.

Statistics

For all statistical analyses, confirm that the following items are present in the figure legend, table legend, main text, or Methods section.

n/a	Confirmed	
[ ]	[x]	The exact sample size (*n*) for each experimental group/condition, given as a discrete number and unit of measurement
[ ]	[x]	A statement on whether measurements were taken from distinct samples or whether the same sample was measured repeatedly
[ ]	[x]	The statistical test(s) used AND whether they are one- or two-sided<br>*Only common tests should be described solely by name; describe more complex techniques in the Methods section.*
[x]	[ ]	A description of all covariates tested
[ ]	[x]	A description of any assumptions or corrections, such as tests of normality and adjustment for multiple comparisons
[ ]	[x]	A full description of the statistical parameters including central tendency (e.g. means) or other basic estimates (e.g. regression coefficient) AND variation (e.g. standard deviation) or associated estimates of uncertainty (e.g. confidence intervals)
[ ]	[x]	For null hypothesis testing, the test statistic (e.g. *F*, *t*, *r*) with confidence intervals, effect sizes, degrees of freedom and *P* value noted<br>*Give P values as exact values whenever suitable.*
[x]	[ ]	For Bayesian analysis, information on the choice of priors and Markov chain Monte Carlo settings
[x]	[ ]	For hierarchical and complex designs, identification of the appropriate level for tests and full reporting of outcomes
[x]	[ ]	Estimates of effect sizes (e.g. Cohen's *d*, Pearson's *r*), indicating how they were calculated

Our web collection on statistics for biologists contains articles on many of the points above.

Software and code

Policy information about availability of computer code

Data collection	CellQuest Pro 6.0<br>NovoExpress v.1.5.0.2001<br>BD FACSDiva Software Version 8.0.1<br>CytExpert 2.4.0.28<br>Quant Studio Real-Time PCR Software v1.7.1<br>IndiGo (Version 2.0.5.0)
Data analysis	GraphPad Prism 9<br>FlowJo V10.8.1<br>R 4.2.1

For manuscripts utilizing custom algorithms or software that are central to the research but not yet described in published literature, software must be made available to editors and reviewers. We strongly encourage code deposition in a community repository (e.g. GitHub). See the Nature Portfolio guidelines for submitting code & software for further information.

Data

Policy information about availability of data

All manuscripts must include a data availability statement. This statement should provide the following information, where applicable:

- Accession codes, unique identifiers, or web links for publicly available datasets
- A description of any restrictions on data availability
- For clinical datasets or third party data, please ensure that the statement adheres to our policy

The RNA-seq data used in this study have been deposited at the Gene Expression Omnibus under accession codes (SuperSeries GEO accession, GSE201977, https://www.ncbi.nlm.nih.gov/geo/query/acc.cgi?acc=GSE201977). The authors declare that the data generated or analyzed during this study are available within the Article, Supplementary Information, or Source Data file. Source data underlying Figs. 1, 2, 3, 4, 5, 6, 7, 8 and supplementary Figs. 1, 2, 3, 4, 5, 6, 7, 8 are provided with this paper. A reporting summary for this article is available as a Supplementary Information file. Raw LC/MS data for cholesterol-targeted metabolomics are not publicly available due to limitations of LipidALL Technologies (http://www.lipidall.com/). Their rationale is that mass spectrometry files contain content that could harm their commercial interests. If necessary, additional information on raw mass spectrometry in this study (Project: 2021-141-C-04) can be requested from LipidALL by email (sales@lipidall.com, qmchu@lipidall.com, smqi@lipidall.com).

Human research participants

Policy information about studies involving human research participants and Sex and Gender in Research.

Reporting on sex and gender	The study did not account for gender. GBM tissue was obtained from six patients, four females and two males.
Population characteristics	Patients with intracranial space-occupying lesions subsequently diagnosed with grade 4 glioma (glioblastoma) were enrolled (n=6). Ages range from 42 to 65 years.
Recruitment	The study involved patients with newly diagnosed primary brain cancer who underwent intracranial surgery for tumor resection and provided consent to donate the resected tissue. A part of the fresh tissue was used for pathology and molecular diagnosis, and the remaining tissue was used for the preparation of single-cell suspension. Finally, a total of 6 GBM patient tumor tissues were collected for analysis. An abstract (translated version) of the approved study protocol has been submitted as Supplementary Note 1.
Ethics oversight	This study has received approval from the Human Research Ethics Committee of the Second Affiliated Hospital of Zhejiang University School of Medicine (ID:2023-0261) and written informed consent was obtained from all participants included in the study. This study performed in accordance with the 1975 Declaration of Helsinki.

Note that full information on the approval of the study protocol must also be provided in the manuscript.

Field-specific reporting

Please select the one below that is the best fit for your research. If you are not sure, read the appropriate sections before making your selection.

☒ Life sciences ☐ Behavioural & social sciences ☐ Ecological, evolutionary & environmental sciences

For a reference copy of the document with all sections, see nature.com/documents/nr-reporting-summary-flat.pdf

Life sciences study design

All studies must disclose on these points even when the disclosure is negative.

Sample size	No statistical method was performed to pre-determine sample sizes, however this was generally determined based on previous studies (Xingzhe Ma. et al., Cell Metabolism, 2019; Sydney R. et al., Nature, 2017; Bo Xu. et al., Nature communications, 2021; Goossens P. et al., Cell Metabolism, 2019).
Data exclusions	No data were excluded from the analyses.
Replication	Biological replicates for each experiment are noted in figure legends.
Randomization	Cell samples or animal tissues were randomly allocated to control (vehicle) and treatment groups (ApoA1, or other treatments) by RANK function.
Blinding	Immunohistochemistry, immunofluorescence, TEM images were acquired and analyzed in a blinded fashion. For in vivo experiments, mice were given a unique number before data collection. The researchers were divided into two groups, one responsible for collecting samples and the other responsible for experiments and data evaluation.

Reporting for specific materials, systems and methods

We require information from authors about some types of materials, experimental systems and methods used in many studies. Here, indicate whether each material, system or method listed is relevant to your study. If you are not sure if a list item applies to your research, read the appropriate section before selecting a response.

Materials & experimental systems

n/a	Involved in the study
☐	☒ Antibodies
☐	☒ Eukaryotic cell lines
☒	☐ Palaeontology and archaeology
☐	☒ Animals and other organisms
☒	☐ Clinical data
☒	☐ Dual use research of concern

Methods

n/a	Involved in the study
☒	☐ ChIP-seq
☐	☒ Flow cytometry
☒	☐ MRI-based neuroimaging

Antibodies

Antibodies used

Antibodies/Source/Identifier/Dilution ratio
Anti-ApoE (EPR19392) Abcam ab183597 1:50
Anti-Hexon Abcam ab252760 1:50
Anti-CD16/CD32 (2.4G2) BD Pharmingen 553142 1:100
Anti-ABCA1 (5A1-1422) BIO-RAD MCA2681 1:100
Anti-OVA (1D3D5) Proteintech 67614-1-Ig 1:1000
Anti-CD45 (30-F11) Biolegend 103112 1:100
Anti-CD45 (HI30) Biolegend 304011 1:100
Anti-CD45.1 (A20) Biolegend 110706 1:100
Anti-CD45.2 (104) Biolegend 109814 1:100
Anti-CD11b (M1/70) Biolegend 101206 1:100
Anti-CD11b (ICRF44) Biolegend 301329 1:100
Anti-F4/80 (BM8) Biolegend 123126 1:100
Anti-CD3 (17A2) Biolegend 100204 1:100
Anti-CD4 (GK1.5) Biolegend 100408 1:100
Anti-CD8 (53-6.7) Biolegend 100732 1:100
Anti-NK1.1 (S17016D) Biolegend 156508 1:100
Anti-CD36 (HM36) Biolegend 102606 1:100
Anti-CD64 (S18017D) Biolegend 161004 1:100
Anti-CD68 (Y1/82A) Biolegend 333813 1:100
Anti-PD-1 (29F.1A12) Biolegend 135206 1:100
Anti-PD-1 (EH12.2H7) Biolegend 329905 1:100
Anti-PD-L1 (10F.9G2) Biolegend 124308 1:100
Anti-Siglec-g (Siglec-10, SH1) Biolegend 163302 1:100
Anti-Siglec-10 (5G6) Biolegend 347603 1:100
Anti-SIRPα (P84) Biolegend 144012 1:100
Anti-LAG-3 (C9B7W) Biolegend 125208 1:100
Anti-TIGIT (4D4/mTIGIT) Biolegend 156104 1:100
Anti-I-A/I-E (MHC-I, M5/114.15.2) Biolegend 107608 1:100
Anti-I-Ab (MHC-II, AF6-120.1) Biolegend 116408 1:100
Anti-TNF-α (MP6-XT22) Biolegend 506306 1:100
Anti-Nos2 (iNOS, W16030C) Biolegend 696806 1:100
Anti-IFN-γ (XMG1.2) Biolegend 505808 1:100
Anti-Gzm-B (QA16A02) Biolegend 372208 1:100
Anti-XBP1 s (9D11A43) Biolegend 658802 1:1000
Anti-CD44 (IM7) Biolegend 103007 1:100
Anti-CD62 L (MEL-14) Biolegend 104406 1:100
Anti-FOXP3 (MF-14) Biolegend 126404 1:100
Alexa Fluor® 488 Donkey anti-rabbit IgG Biolegend 406416 1:100
InVivo Mab rat IgG1 Isotype control (TNP6A7) BioXCell BP0290 10 mg/kg
InVivo Mab Anti-mouse CD4 (GK1.5) BioXCell BE0003-1 10 mg/kg
InVivo Mab Anti-mouse CD8α (YTS 169.4) BioXCell BE0117 10 mg/kg
InVivo Mab Anti-mouse NK1.1 (PK136) BioXCell BE0036 10 mg/kg
InVivo Mab Anti-mouse CSF1R (AFS98) BioXCell BE0213 10 mg/kg
Anti-SREBP2 Proteintech 28212-1-AP 1:1000
Anti-LDLR Proteintech 10785-1-AP 1:50 (IF), 1:1000 (WB)

Anti-β-Actin (2D4H5) Proteintech 66009-1-Ig 1:1000
Anti-ApoA1 Proteintech 14427-1-AP 1:1000
Anti-ABCG1 Proteintech 13578-1-AP 1:100 (FC), 1:1000 (WB)
Anti-NeuN Servicebio GB11138 1:50
Anti-GFAP Servicebio GB11096 1:50
Anti-EGFR Sangon Biotech D160292 1:1000
Anti-ABCA1 (HJ1) Abcam ab66217 1:100 (FC), 1:1000 (WB)
Anti-F4/80 MicroBeads UltraPure, mouse Miltenyi Biotec 130-110-443 1:10
CD11b MicroBeads UltraPure, mouse Miltenyi Biotec 130-126-725 1:10
CD8a (Ly-2) MicroBeads, mouse Miltenyi Biotec 130-117-044 1:10
CD14 MicroBeads, human Miltenyi Biotec 130-050-201 1:10
T-Cell Activation/Expansion Kit, mouse Miltenyi Biotec 130-093-627 bead-to-cell ratio 1:1

Validation

The primary antibodies have been validated their respective manufacturers for their respective species, and applications. The validation statement for each primary antibody is available on the manufacturer's website. In some experiments, we used an isotype control or negative and positive samples as controls.
Antibodies/Source/Identifier/Dilution ratio
Anti-ApoE (EPR19392) Abcam ab183597:
Species: Mouse, Rat, Human; Application: Flow Cyt (Intra), IP, ICC/IF, IHC-P, WB; Manufacturer's website: https://www.abcam.cn/products/primary-antibodies/apolipoprotein-e-antibody-epr19392-ab183597.html

Anti-Hexon Abcam ab252760:Application:
Species: Tissue, cells or virus corresponding to Human Adenovirus hexon protein. Adenovirus type 3 (ATCC strain VR847); Application: IF; Validated in this paper.

Anti-CD16/CD32 (2.4G2) BD Pharmingen 553142:
Species: Mouse; Application: Blocking, Flow cytometry (Routinely Tested), Immunohistochemistry-frozen (Tested During Development), Immunoprecipitation (Reported); Manufacturer's website: https://www.bdbiosciences.com/en-us/products/reagents/flow-cytometry-reagents/research-reagents/single-color-antibodies-ruo/purified-rat-anti-mouse-cd16-cd32-mouse-bd-fc-block.553142

Anti-ABCA1 (5A1-1422) BIO-RAD MCA2681:
Species: Mouse; Application: Flow cytometry; Manufacturer's website: https://www.bio-rad-antibodies.com/monoclonal/mouse-abca1-antibody-5a1-1422-mca2681.html
f=purified&JSESSIONID_STERLING=430B0E29D30955ECAA0D42B01AB0CBE4.ecommerce1&evCntryLang=CN-zh&cntry=CN&thirdPartyCookieEnabled=true

Anti-OVA (1D3D5) Proteintech 67614-1-Ig:
Species: Chicken; Application: WB, ELISA; Manufacturer's website: https://www.ptglab.co.jp/Products/Ovalbumin-Antibody-67614-1-Ig.htm

Anti-CD45 (30-F11) Biolegend 103112:
Species: Mouse; Application: Flow cytometry; Manufacturer's website: https://www.biolegend.com/en-us/products/apc-anti-mouse-cd45-antibody-97

Anti-CD45 (HI30) Biolegend 304011:
Species: Human; Application: Flow cytometry; Manufacturer's website: https://www.biolegend.com/en-us/products/apc-anti-human-cd45-antibody-705

Anti-CD45.1 (A20) Biolegend 110706:
Species: Mouse; Application: Flow cytometry; Manufacturer's website: https://www.biolegend.com/en-us/products/fitc-anti-mouse-cd45-1-antibody-198

Anti-CD45.2 (104) Biolegend 109814:
Species: Mouse; Application: Flow cytometry; Manufacturer's website: https://www.biolegend.com/en-us/products/apc-anti-mouse-cd45-2-antibody-2759

Anti-CD11b (M1/70) Biolegend 101206:
Species: Mouse, Human, Cynomolgus, Rhesus; Application: Flow cytometry; Manufacturer's website: https://www.biolegend.com/en-us/products/fitc-anti-mouse-human-cd11b-antibody-347

Anti-CD11b (ICRF44) Biolegend 301329:
Species: Human, Cynomolgus, Rhesus; Application: Flow cytometry; Manufacturer's website: https://www.biolegend.com/en-us/products/fitc-anti-human-cd11b-antibody-8299

Anti-F4/80 (BM8) Biolegend 123126:
Species: Mouse; Application: Flow cytometry; Manufacturer's website: https://www.biolegend.com/en-us/products/percp-anti-mouse-f4-80-antibody-4302

Anti-CD3 (17A2) Biolegend 100204:

Species: Mouse; Application: Flow cytometry; Manufacturer's website: https://www.biolegend.com/en-us/products/fitc-anti-mouse-cd3-antibody-45

Anti-CD4 (GK1.5) Biolegend 100408:
Species: Mouse; Application: Flow cytometry; Manufacturer's website: https://www.biolegend.com/en-us/products/pe-anti-mouse-cd4-antibody-250

Anti-CD8 (53-6.7) Biolegend 100732:
Species: Mouse; Application: Flow cytometry; Manufacturer's website: https://www.biolegend.com/en-us/products/percp-anti-mouse-cd8a-antibody-4256

Anti-NK1.1 (S17016D) Biolegend 156508:
Species: Mouse; Application: Flow cytometry; Manufacturer's website: https://www.biolegend.com/en-us/products/fitc-anti-mouse-nk-11-antibody-19869

Anti-CD36 (HM36) Biolegend 102606:
Species: Mouse; Application: Flow cytometry; Manufacturer's website: https://www.biolegend.com/en-us/products/pe-anti-mouse-cd36-antibody-2675

Anti-CD64 (S18017D) Biolegend 161004:
Species: Mouse; Application: Flow cytometry; Manufacturer's website: https://www.biolegend.com/en-us/products/pe-anti-mouse-cd64-fc%ce%b3ri-antibody-21518

Anti-CD68 (Y1/82A) Biolegend 333813:
Species: Human; Application: Flow cytometry; Manufacturer's website: https://www.biolegend.com/en-us/products/percp-cyanine5-5-anti-human-cd68-antibody-7007

Anti-PD-1 (29F.1A12) Biolegend 135206:
Species: Mouse; Application: Flow cytometry; Manufacturer's website: https://www.biolegend.com/en-us/products/pe-anti-mouse-cd279-pd-1-antibody-6170

Anti-PD-1 (EH12.2H7) Biolegend 329905:
Species: Human; Application: Flow cytometry; Manufacturer's website: https://www.biolegend.com/en-us/products/pe-anti-human-cd279-pd-1-antibody-4412

Anti-PD-L1 (10F.9G2) Biolegend 124308:
Species: Mouse; Application: Flow cytometry; Manufacturer's website: https://www.biolegend.com/en-us/products/pe-anti-mouse-cd274-b7-h1-pd-l1-antibody-4497

Anti-Siglec-g (Siglec-10, SH1) Biolegend 163302:
Species: Mouse; Application: Flow cytometry; Manufacturer's website: https://www.biolegend.com/en-us/products/purified-anti-mouse-siglec-g-antibody-20651

Anti-Siglec-10 (5G6) Biolegend 347603 1:100
Species: Human; Application: Flow cytometry; Manufacturer's website: https://www.biolegend.com/en-us/products/pe-anti-human-siglec-10-antibody-6431

Anti-SIRPα (P84) Biolegend 144012:
Species: Mouse; Application: Flow cytometry; Manufacturer's website: https://www.biolegend.com/en-us/products/pe-anti-mouse-cd172a-sirpalpha-antibody-9801

Anti-LAG-3 (C9B7W) Biolegend 125208:
Species: Mouse; Application: Flow cytometry; Manufacturer's website: https://www.biolegend.com/en-us/products/pe-anti-mouse-cd223-lag-3-antibody-4486

Anti-TIGIT (4D4/mTIGIT) Biolegend 156104:
Species: Mouse; Application: Flow cytometry; Manufacturer's website: https://www.biolegend.com/en-us/products/pe-anti-mouse-tigit-vstm3-antibody-16484

Anti-I-A/I-E (MHC-I, M5/114.15.2) Biolegend 107608:
Species: Mouse; Application: Flow cytometry; Manufacturer's website: https://www.biolegend.com/en-us/products/pe-anti-mouse-i-a-i-e-antibody-367

Anti-I-Ab (MHC-II, AF6-120.1) Biolegend 116408:
Species: Mouse; Application: Flow cytometry; Manufacturer's website: https://www.biolegend.com/en-us/products/pe-anti-mouse-i-ab-antibody-1741

Anti-TNF-α (MP6-XT22) Biolegend 506306:
Species: Mouse; Application: Flow cytometry; Manufacturer's website: https://www.biolegend.com/en-us/products/pe-anti-mouse-tnf-alpha-antibody-978

Anti-Nos2 (iNOS, W16030C) Biolegend 696806:
Species: Mouse; Application: Flow cytometry; Manufacturer's website: https://www.biolegend.com/en-us/products/pe-anti-nos2-inos-antibody-19910

Anti-IFN-γ (XMG1.2) Biolegend 505808:
Species: Mouse; Application: Flow cytometry; Manufacturer's website: https://www.biolegend.com/en-us/products/pe-anti-mouse-ifn-gamma-antibody-997

Anti-Gzm-B (QA16A02) Biolegend 372208:
Species: Human, Mouse; Application: Flow cytometry; Manufacturer's website: https://www.biolegend.com/en-us/products/pe-anti-human-mouse-granzyme-b-recombinant-antibody-14431

Anti-XBP1 s (9D11A43) Biolegend 658802:
Species: Mouse; Application: Western blotting, ChIP, IP; Manufacturer's website: https://www.biolegend.com/en-us/products/purified-anti-xbp-1s-antibody-9139

Anti-CD44 (IM7) Biolegend 103007:
Species: Human, Mouse; Application: Flow cytometry; Manufacturer's website: https://www.biolegend.com/en-us/products/pe-anti-mouse-human-cd44-antibody-2206

Anti-CD62 L (MEL-14) Biolegend 104406:
Species: Mouse; Application: Flow cytometry; Manufacturer's website: https://www.biolegend.com/en-us/products/fitc-anti-mouse-cd62l-antibody-384

Anti-FOXP3 (MF-14) Biolegend 126404:
Species: Mouse; Application: Flow cytometry; Manufacturer's website: https://www.biolegend.com/en-us/products/pe-anti-mouse-foxp3-antibody-4660

Alexa Fluor® 488 Donkey anti-rabbit IgG Biolegend 406416:
Species: Rabbit; Application: FC - Quality tested; Manufacturer's website: https://www.biolegend.com/en-us/products/alexa-fluor-488-donkey-anti-rabbit-igg-minimal-x-reactivity-9380

InVivo Mab rat IgG1 Isotype control (TNP6A7) BioXCell BP0290:
Species: Rabbit; Application: an isotype-matched control for rat IgG1 antibodies in most in vivo and in vitro applications; Manufacturer's website: https://bioxcell.com/invivoplus-rat-igg1-isotype-control-anti-trinitrophenol

InVivo Mab Anti-mouse CD4 (GK1.5) BioXCell BE0003-1
Species: Mouse; Application: in vivo CD4+ T cell depletion, Flow cytometry, Western blot; Manufacturer's website: https://bioxcell.com/invivomab-anti-mouse-cd4-be0003-1

InVivo Mab Anti-mouse CD8α (YTS 169.4) BioXCell BE0117:
Species: Mouse; Application: in vivo CD8+ T cell depletion, Western blot; Manufacturer's website: https://bioxcell.com/invivomab-anti-mouse-cd8a-be0117

InVivo Mab Anti-mouse NK1.1 (PK136) BioXCell BE0036:
Species: Mouse; Application: n vivo NK cell depletion, Flow cytometry; Manufacturer's website: https://bioxcell.com/invivomab-anti-mouse-nk1-1-be0036

InVivo Mab Anti-mouse CSF1R (AFS98) BioXCell BE0213:
Species: Mouse; Application: in vivo macrophage depletion, in vitro CSF1R neutralization, in vivo monocyte depletion, Flow cytometry, Western blot; Manufacturer's website: https://bioxcell.com/invivomab-anti-mouse-csf1r-cd115-be0213

Anti-SREBP2 Proteintech 28212-1-AP:
Species: Hamster, Human, Mouse; Application: WB, IHC, ELISA; Manufacturer's website: https://www.ptglab.co.jp/products/SREBF2-Antibody-28212-1-AP.htm

Anti-LDLR Proteintech 10785-1-AP:
Species: Chicken, Hamster, Human, Mouse, Pig, Rat; Application: WB, IP, IHC, IF, FC, ELISA; Manufacturer's website: https://www.ptglab.co.jp/products/LDLR-Antibody-10785-1-AP.htm

Anti-β-Actin (2D4H5) Proteintech 66009-1-Ig:
Species: Human, Mouse, Rat, Hamster, Monkey, Dog, Pig, Chicken, Rabbit, Zebrafish; Application: WB, IP, IHC, IF, FC, CoIP, ChIP, ELISA; Manufacturer's website: https://www.ptglab.co.jp/products/Pan-Actin-Antibody-66009-1-Ig.htm

Anti-ApoA1 Proteintech 14427-1-AP:
Species: Human, Mouse; Application: WB, IP, IHC, IF, FC, ELISA; Manufacturer's website: https://www.ptglab.co.jp/products/APOA1-Antibody-14427-1-AP.htm

Anti-ABCG1 Proteintech 13578-1-AP:

Species: Human, Mouse, Rat; Application: WB, IP, IHC, IF, ELISA; Manufacturer's website: https://www.ptglab.co.jp/products/ABCG1-Antibody-13578-1-AP.htm

Anti-NeuN Servicebio GB11138:
Species: Human, Mouse, Rat; Application: WB, IHC/IF; Manufacturer's website: https://www.servicebio.cn/goodsdetail?id=1406

Anti-GFAP Servicebio GB11096:
Species: Human, Mouse, Rat; Application: WB, IHC/IF; Manufacturer's website: https://www.servicebio.cn/goodsdetail?id=1376

Anti-EGFR Sangon Biotech D160292:
Species: Human, Mouse; Application: WB, IHC/IF; Manufacturer's website: https://www.sangon.com/productDetail?productInfo.code=D160292

Anti-ABCA1 (HJ1) Abcam ab66217 1:100 (FC), 1:1000 (WB)
Species: Mouse, Rat, Human; Application: WB, Flow Cyt, IHC-P; Manufacturer's website: https://www.abcam.cn/products/primary-antibodies/abca1-antibody-hj1-ab66217.html

Anti-F4/80 MicroBeads UltraPure, mouse Miltenyi Biotec 130-110-443:
Species: Mouse; Application: isolation of mouse macrophages; Manufacturer's website: https://www.miltenyibiotec.com/US-en/products/anti-f4-80-microbeads-ultrapure-mouse.html#130-110-443

CD11b MicroBeads UltraPure, mouse Miltenyi Biotec 130-126-725:
Species: Mouse; Application: isolation or depletion of mouse cells based on their CD11b expression; Manufacturer's website: https://www.miltenyibiotec.com/US-en/products/cd11b-microbeads-ultrapure-mouse.html#130-126-725

CD8a (Ly-2) MicroBeads, mouse Miltenyi Biotec 130-117-044:
Species: Mouse; Application: positive selection or depletion of mouse CD8a+ T cells; Manufacturer's website: https://www.miltenyibiotec.com/US-en/products/cd8a-ly-2-microbeads-mouse.html#130-117-044

CD14 MicroBeads, human Miltenyi Biotec 130-050-201:
Species: Human; Application: selection or depletion of human monocytes and macrophages; Manufacturer's website: https://www.miltenyibiotec.com/US-en/products/cd14-microbeads-human.html#130-050-201

T-Cell Activation/Expansion Kit, mouse Miltenyi Biotec 130-093-627:
Species: Mouse; Application: activation and expansion of mouse T cells; Manufacturer's website: https://www.miltenyibiotec.com/US-en/products/t-cell-activation-expansion-kit-mouse.html#130-093-627

Eukaryotic cell lines

Policy information about cell lines and Sex and Gender in Research

Cell line source(s)	293T (RRID: CVCL_0063) ATCC CRL-3216<br>U138-MG (RRID: CVCL_0020) ATCC HTB-16<br>T98G (RRID: CVCL_0556) Procell CL-0583<br>U251-MG (RRID: CVCL_0021) CCTCC GDC0093<br>THP-1 (RRID: CVCL_0006) CCTCC GDC0100<br>GL261 (RRID: CVCL_Y003) BCRJ 0299<br>G422 NICR PUMC000314<br>MC38 (RRID: CVCL_B288) NICR PUMC000523<br>C6 (RRID: CVCL_0194) NICR PUMC000131<br>BMDMs was obtained based on a previous report (Hagemann T. et al., J Exp Med, 2008)
Authentication	Human cell lines were authenticated by short tandem repeat (STR) analysis. Murine cell lines were authenticated through a large number of experiments, such as morphology, immunocompetent tumor transplantation model, immunohistochemical staining of glial cell marker GFAP, etc.
Mycoplasma contamination	Mycoplasma contamination was detected by Myco-Lumi™ (Beyotime). No mycoplasma contamination was observed.
Commonly misidentified lines (See ICLAC register)	No cell lines listed in the ICLAC database were used.

Animals and other research organisms

Policy information about studies involving animals; ARRIVE guidelines recommended for reporting animal research, and Sex and Gender in Research

Laboratory animals	Four-to-six week old male Balb/c-Nude, six-to-eight week old male C57BL/6J mice, NOD/ShiLtJGpt-Prkdcem26Cd52Il2rgem26Cd22/Gpt (NCG) mice, and B6/JGpt-Ptprcem1Cin(p. K302E)/Gpt (CD45.1) mice were purchased from the Model Animal Research Center of Nanjing University. Six-to-eight week old male C57BL/6-Tg (TcraTcrb) 1100Mjb/J (OT-I) mice were purchased from the Jackson Laboratory. Six-to-eight week old male KM mice and SD rats were purchased from Nanjing Junke Biotechnology Co., Ltd., China.

	Animals were housed in SPF facilities at the Medical School of Nanjing University. Housing conditions were as follows: the light time was 8:00-20:00, the temperature was 18-22°C, and the relative humidity was 40-70%. Animal care and handling procedures were in accordance with the NIH Guide for the Care and Use of Laboratory Animals and were approved by the Nanjing University Institutional Review Board.<br>Rhesus monkeys were purchased from Hubei Tianqin Biotechnology Co., Ltd. Suizhou Branch (1)/Xinye County Xinyu Wildlife Breeding Co., Ltd. (2). Animal certificate number: 421218300000162(1)/410934211100000964(2)/410934211100001081(2). Monkeys were housed in InnoStar GLP laboratory according to NMPA (CFDA) guidelines. Both female and male animals were used in the preclinical safety studies. The study protocol was approved by the Institutional Animal Care and Use Committee of Shanghai InnoStar Bio-Tech Co. Ltd. (IACUC No.: IACUC-2021-M-072; 21091RD02). Both female and male animals were used in the preclinical safety studies.
Wild animals	The study did not involve wild animals.
Reporting on sex	Biological activity investigations are based on male animals. Rhesus monkey toxicity studies are divided between male and female.
Field-collected samples	The study did not involve samples collected from the field.
Ethics oversight	Animal Welfare: This study will comply with all applicable sections of the "Guide for the Care and Use of Laboratory Animals" (2011) issued by the National Research Council, USA, "Laboratory Animal Administration" (2017) issued by the State Science and Technology Committee, People's Republic of China, and "Laboratory Animal Administration Regulations" (1997) issued by the Shanghai Laboratory Animal Administration Office, P. R. China. The protocol, amendment(s), and procedures involving the care and use of animals in the study will be reviewed and approved by the Institutional Animal Care and Use Committee (IACUC) of the test facility, including the Nanjing University Institutional Review Board.

Note that full information on the approval of the study protocol must also be provided in the manuscript.

Flow Cytometry

Plots

Confirm that:

- [x] The axis labels state the marker and fluorochrome used (e.g. CD4-FITC).
- [x] The axis scales are clearly visible. Include numbers along axes only for bottom left plot of group (a 'group' is an analysis of identical markers).
- [x] All plots are contour plots with outliers or pseudocolor plots.
- [x] A numerical value for number of cells or percentage (with statistics) is provided.

Methodology

Sample preparation	Brain tumor samples were minced in 2% RPMI 1640 medium containing 0.5 mg/ml collagenase type IV and digested at 37°C for 30 min. Cells were further separated with 70 μm cell strainers and resuspended in excess of complete RPMI 1640 medium. Cells were centrifuged at 400 × g for 5 min to obtain a single-cell suspension. Before staining, the cell suspensions were incubated with an Fc blocker (anti-CD16/32) at 4°C for 20 min. The cell suspensions were then stained with PI (5 μg/ml) at room temperature for 5 min to determine cell viability. For cell surface staining, cell suspensions were incubated with the indicated antibodies (0.25 μg/106 cells) in 100 μl FACS buffer at 4°C for 30 min. For intracellular staining, cells were stimulated with PMA and Ionomycin, and brefeldin A (MCE, HY-16592) was added to the cultures for 5 h to block cytokine secretion. Cell suspensions were fixed in Fix/Perm buffer at 4°C for 30 min and then incubated with Perm buffer at 4°C for 45 min. Cell suspensions were stained with intracellular antibodies at 4°C for 30 min. For CFSE cell division, T cells were incubated with 5 μM CFSE working solution for 20 min at room temperature. Cells were incubated with 10% FBS medium for 10 min to quench staining. CFSE-labeled cells were subjected to downstream analysis.
Instrument	Samples were analyzed using BD FACS Calibur, BD Aria I, Beckman CytoFLEX LX, ACEA Novacyte, or Gaugene MoniSight 820.
Software	Data collection:<br>CellQuest Pro 6.0<br>NovoExpress v.1.5.0.2001<br>BD FACSDiva Software Version 8.0.1<br>CytExpert 2.4.0.28<br>Data analysis:<br>Data were analyzed with FlowJo v10.8.1.
Cell population abundance	Sorting purity of TAMs was evaluated by acquisition of sorted fractions directly after the sort using the same gates for sorting. Purities were generally above 90%.
Gating strategy	Gating strategy for the difference in phagocytosis between PD-1+ TAMs and PD-1-TAMs is shown in Extended Data Fig. 1c. Briefly, cells were gated on FSC-A/SSC-A. Doublets were excluded by FSC-H/FSC-A. Alive immune cells were gated on CD45 positive and PI negative cell fraction. TAMs were gated on CD11-b positive and F4-80 positive cell fraction. Gating for PD-1-TAMs were determined using isotype control, and PD-1+ TAMs were determined using anti-PD-1-PE-Cy7. Phagocytosis of TAMs was gated on YFP positive cell fraction. Cholesterol level of TAMs was determined by Filipin-III stain. Similarly, gating strategy for Siglec-10 TAMs can be obtained.<br>Gating strategy for the cell-cell contact between TAMs and OT-I T cells, cells were gated on FSC-H/SSC-H. OT-I T cells were

gated on F4-80 negative and SSC-H low cell fraction. TAMs were gated on F4-80 positive and SSC-H medium to high cell fraction. The cell-cell contact were gated on CD3 positive and CFSE positive TAMs. Singlets and doublets was further determined by FSC-H.

Gating strategy for the exhaustion and activation of CD8+ T cells, cell were gated on FSC-H/SSC-H. Immune cells were gated on CD45 positive cell fraction. CD8+ T cells were gated on SSC-H low, CD8 positive, and CD3 positive cell fraction. CD8+ T cells with surface markers such as PD-1, LAG-3, TIGIT, CD44, CD62L, et. positive and intracellular markers such as IFN-γ and Gzm-B positive were gated.

☒ Tick this box to confirm that a figure exemplifying the gating strategy is provided in the Supplementary Information.
